# Hypoxia-inducible factor-2α is crucial for proper brain development

**DOI:** 10.1038/s41598-020-75838-4

**Published:** 2020-11-05

**Authors:** Kira Kleszka, Tristan Leu, Theresa Quinting, Holger Jastrow, Sonali Pechlivanis, Joachim Fandrey, Timm Schreiber

**Affiliations:** 1 Institute of Physiology, University Duisburg-Essen, Essen, Germany; 2 Institute of Anatomy and Institute for Experimental Immunology and Imaging, Imaging Centre Essen, Electron Microscopy Unit, Essen University Hospital, Essen, Germany; 3 Institute for Medical Informatics, Biometry and Epidemiology, University Hospital of Essen, University Duisburg-Essen, Essen, Germany; 4 Department of Physiology, Pathophysiology and Toxicology and Center for Biomedical Education and Research (ZBAF), University of Witten/Herdecke, 58453 Witten, Germany

**Keywords:** Physiology, Neurophysiology, Developmental neurogenesis

## Abstract

Sufficient tissue oxygenation is required for regular brain function; thus oxygen supply must be tightly regulated to avoid hypoxia and irreversible cell damage. If hypoxia occurs the transcription factor complex hypoxia-inducible factor (HIF) will accumulate and coordinate adaptation of cells to hypoxia. However, even under atmospheric O_2_ conditions stabilized HIF-2α protein was found in brains of adult mice. Mice with a neuro-specific knockout of *Hif-2α* showed a reduction of pyramidal neurons in the retrosplenial cortex (RSC), a brain region responsible for a range of cognitive functions, including memory and navigation. Accordingly, behavioral studies showed disturbed cognitive abilities in these mice. In search of the underlying mechanisms for the specific loss of pyramidal cells in the RSC, we found deficits in migration in neural stem cells from *Hif-2α *knockout mice due to altered expression patterns of genes highly associated with neuronal migration and positioning.

## Introduction

Oxygen is essential for most life on earth and affects various life activities including growth and development. Although oxygen is vital to maintain normal function of all organs, oxygen levels in the tissue are always substantially lower than the pO_2_ in the air we breathe^1^. Especially in the brain, which critically depends on oxygen supply^2^, oxygen levels in almost all regions are rather low in the adult (pO_2_ of 11.4—53.2 mmHg) or even lower in the fetal brain^3^ (0.076—7.6 mmHg). Thus, oxygen supply must precisely match the local demand to prevent critical hypoxia with irreversible brain damage^2^.

Hypoxia initiates a wide range of cellular responses to maintain oxygen supply, including altered gene expression to optimize supply and adapt metabolism. 89% of hypoxia-inducible genes appear to have a common mode of regulation that involves activation of hypoxia-inducible factor (HIF), an oxygen-responsive member of the helix-loop-helix PAS (PER-ARNT-SIM) family^4^. HIF is a heterodimer composed of an oxygen-sensitive α-subunit (HIFα) and a constitutive β-subunit (HIF-1β, also known as ARNT). Three HIFα subunits have been reported: while HIF-1α or HIF-2α (EPAS1) dimerizes with HIF-1β to drive hypoxia-induced target gene expression, the function of HIF-3α is less obvious^5^. In normoxic conditions, prolyl hydroxylases (PHD-1—PHD-3) and von Hippel-Lindau protein (VHL) target HIF-1/2α for proteasomal degradation^6,7^. Under hypoxia, oxygen-dependent PHD activity ceases and HIFαs accumulate due to reduced degradation.

*Hif-1α*, *Hif-2α* and *Hif-1β* are expressed in the developing brain, and modulate gene activity in response to low oxygen in the brain *in vivo*^8^. Neural cell-specific *Hif-1α* deficient mice exhibit a hydrocephalus accompanied by a reduction of neuronal cells and an impairment of spatial memory, indicating that HIF is crucial for brain development^9^. However, the role of HIF-2α during neural development is still not well understood. Duan et al*.* showed that a loss of HIF-2α in astrocytes leads to disturbed astrogenesis in the murine retina^10^. In zebrafish, HIF-2α protects neural progenitor cells and neural differentiation processes by up-regulating the survival orthologues Birc5a and Birg5b during embryogenesis^11^. A recent study investigating the role of astrocytic HIF-1α and HIF-2α in synaptic plasticity found that loss of HIF-2α could affect cognitive performance in mice^12^.

The aim of our study was to investigate the role of HIF-2 in the murine brain. Since we found HIF-2α protein already stabilized in the normoxic brain of wild type animals, we bred a neural specific *Hif-2α* knockout mouse to investigate the role of HIF-2 in normal brain function with a focus on development.

## Results

### HIF-2α is stabilized in the normoxic mouse brain

**Figure 1:**
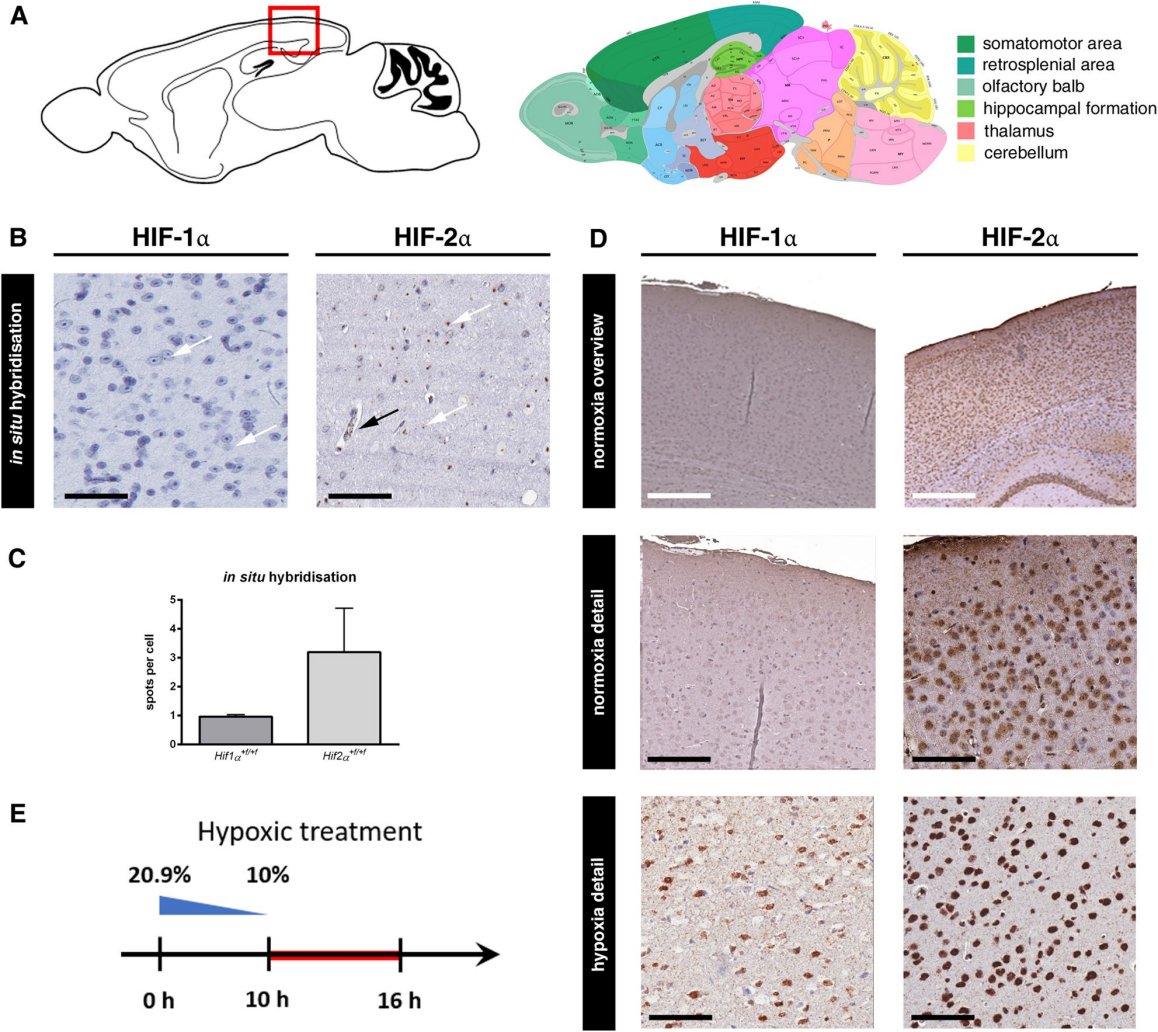
HIF-2α is stabilised in the normoxic mouse brain. (**A)** Schema of anatomical region of the retrosplenial cortex in adult mice (image credit: Allen Mouse Brain Atlas). (**B, C)** In situ hybridisation and quantification of *Hif-1α* and *Hif-2α* mRNA in wild type mouse brains in endothelial (black arrow) and neural cells (white arrows). (**D)** Immunohistochemical staining of HIF-1α and HIF-2α (brown) in wild type brains under normoxic and hypoxic conditions. Representative photomicrographs are shown. (**E)** Timeline of hypoxic treatment to induce HIF-1α stabilisation. Oxygen concentration was reduced from 20.9 to 10% over 10 h and remained at this point for additional 6 h. Scale bars: 100 µm (black), x (white). Data is representative for experiments with at least three mice.

To determine the role of HIF in the brain, we quantified *Hif-1α* and *Hif-2α* expression in the adult brain (P70) using in situ hybridization (Fig. 1A). *Hif-1α* mRNA was ubiquitously expressed and equally distributed in all brain cells. *Hif-2α* was strongly expressed in endothelial cells of blood vessels, but was also present in all other cell types of the brain, including cells in adult stem cell niches, like the subventricular and subgranular zone. Moreover, expression of *Hif-2α* was significantly higher than *Hif-1α* (Fig. 1B and C). Because HIF-α is acutely regulated on the protein level by hypoxic post-translational stabilization, we determined protein levels using immunohistochemistry (Fig. 1D). To our surprise, HIF-2α protein was found throughout the brain in conventionally housed animals kept under atmospheric O_2_ conditions. In contrast, HIF-1α protein was not detected in the normoxic brain and was only stabilized after hypoxic treatment (Fig. 1D and E). Thus, we found HIF-2α constantly expressed and stabilized in the normoxic adult mouse brain.

### Hif-2α knockout in the brain leads to a significant loss of pyramidal cells in the retrosplenial cortex

**Figure 2:**
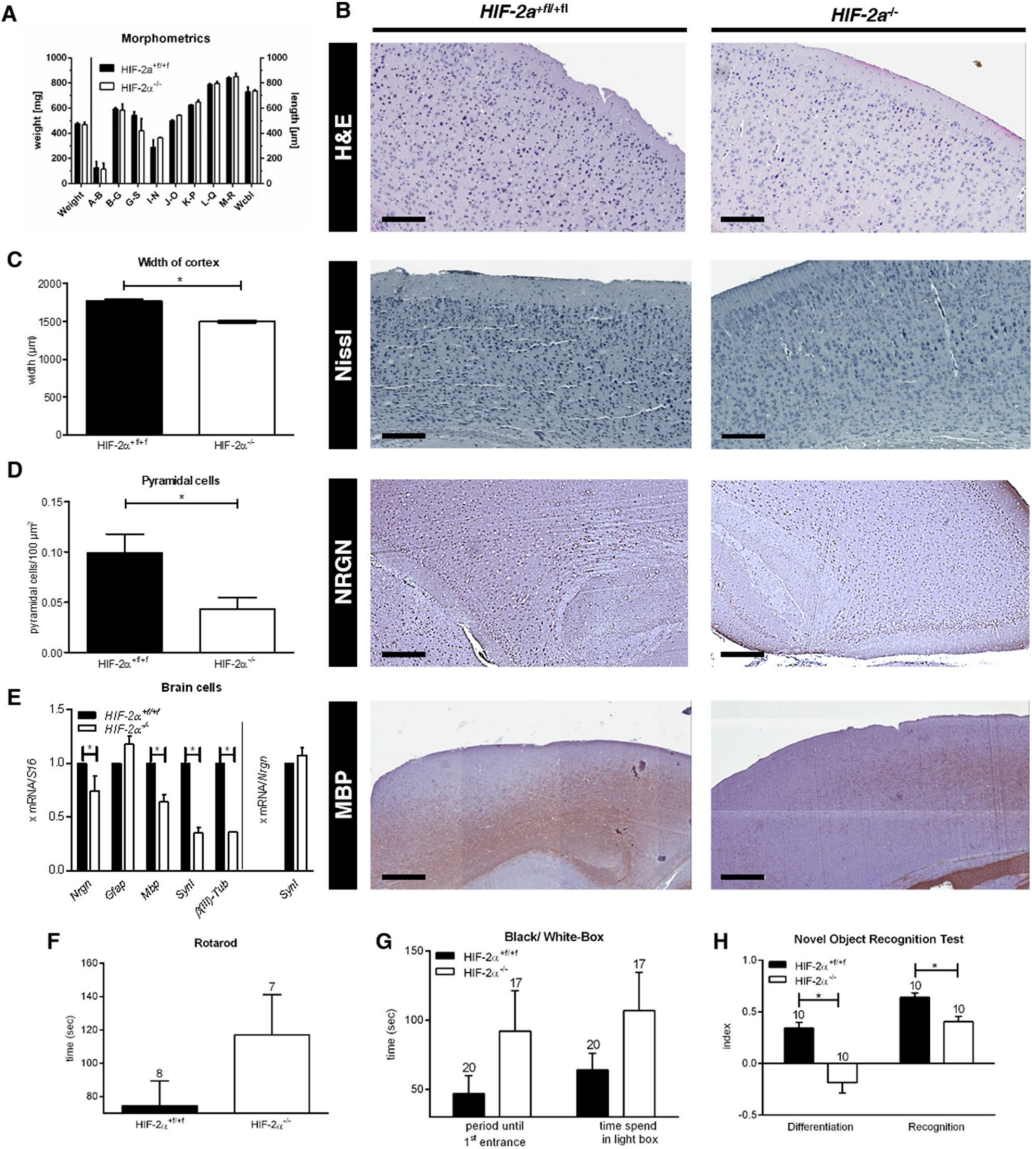
*HIF-2α* knockout in the brain leads to a significant loss of pyramidal cells in the retrosplenial cortex. (**A)** Brain weight and morphometrics according to Shimada et al*.*^13^. (**B)** H&E, Nissl, and immunohistochemical (brown) staining of murine cortices of *Hif-2α*^*f*+*/f*+^ and *Hif-2α*^*-/-*^ animals. (**C)** Maximum width measurement of the prefrontal cortex. (**D)** Quantification of NRGN immunoreactive cells of the IHC staining of the retrosplenial area. (**E)** Real-time PCR analyses of *Neurogranin* (*Nrgn*, pyramidal cells), *Glial fibrillary protein* (*Gfap*, astrocytes), *Myelin basic protein* (*Mbp*, oligodendrocytes), *Synapsin 1* (*Synapsin*, presynapses) and *b(III) Tubulin* (*Tubulin*, neurons). (**F–H)** Behavioural studies. The number above each bar indicates the animals used for analysis. Scale bars: 200 µm. Data is representative for experiments with at least three mice. Each column represents the mean value ± SE. *p < 0.05 compared as indicated.

As HIF-2α was constantly present in the brain, it likely has an important role in normal brain function. To investigate the impact during brain development, we created a conditional brain specific *Hif-2α* knockout mouse by crossing mice with double-floxed *Hif-2α* (exon 2 flanked by loxP sites) with mice heterozygous for *Cre recombinase* under control of the *Nestin* promoter. Thus, *Hif-2α*^+*f/*+*f*^ x *Nes-Cre*^+*/-*^ mice have a dysfunctional HIF-2α protein lacking the DNA-binding domain in neural progenitor and all descending cells (Suppl. Figure s1). *Hif-2α*^+*f/*+*f*^ x *Nes-Cre*^+*/-*^ animals, in the following termed *Hif-2α*^*-/-*^, had a normal life span and fertility, and showed no obvious neurological deficits. Brain weight and morphometrics^13^ did not differ from wild type (*Hif-2α*^+*f/*+*f*^) littermates (Fig. 2A). In H&E staining of sagittal sections the maximal cortex width was slightly reduced in *Hif-2α*^*-/-*^ animals compared to wild type mice (Fig. 2B and C). Additionally, counts on Nissl stained sections revealed a reduced number of pyramidal cells in the retrosplenial cortex (RSC) by more than 50% (Fig. 2D). In contrast, pyramidal cells in the prefrontal cortex (PFC) and the hippocampus were not affected (data not shown). The loss of pyramidal cells was confirmed by immunohistochemistry, mRNA, and protein analysis for neurogranin (Fig. 2B and 2E, Suppl. Figure s2). Moreover, we found a reduction in *myelin basic protein* (*Mbp*) and *synapsin 1* (*Syn1*) expression in the cortex of *Hif-2α*^*-/-*^ mice (Fig. 2B and 2E). We also looked for morphological changes in other cell types that express *Nestin* during their development, like pericytes and endothelial cells, but did not find any substantial differences.

### Hif-2α knockout mice have impaired learning, memory, and fear induction

The RSC is key in a core network of brain regions important for several cognitive functions. Moreover, the RSC is regularly compromised in common neurological disorders that impair memory^14^. We performed different tests to investigate, if the observed alterations in the RSC lead to disturbances in motor abilities (Rotarod), behavior (light–dark box), and learning and memory (novel object recognition test, NOR). In the Rotarod test, *Hif-2α*^*-/-*^ mice showed slightly enhanced motor skills (Fig. 2F). The light–dark box indicated a reduced fear-associated behavior in the knockout animals (Fig. 2G), which spent nearly twice as much time in the lighted department, which is typically avoided. However, results in both tests were not statistically significant due to high individual differences in both groups. Interestingly, the NOR test showed diminished abilities in learning and memory after loss of HIF-2α (Fig. 2H). The *differentiation index* represents the capability to distinguish between two different objects, respectively the capability to remember the old object and identify the new one, which relates to learning, whereas the *recognition index* characterizes memory function. Both indices revealed significantly poorer competences of *Hif-2α*^*-/-*^ mice compared to wild type animals. In conclusion our results demonstrate that loss of HIF-2α during brain development leads to loss of predominantly pyramidal cells in the RSC, accompanied by severe impairments in learning and memory.

### Loss of HIF-2α in NSC affects various parameters during neural development

**Figure 3:**
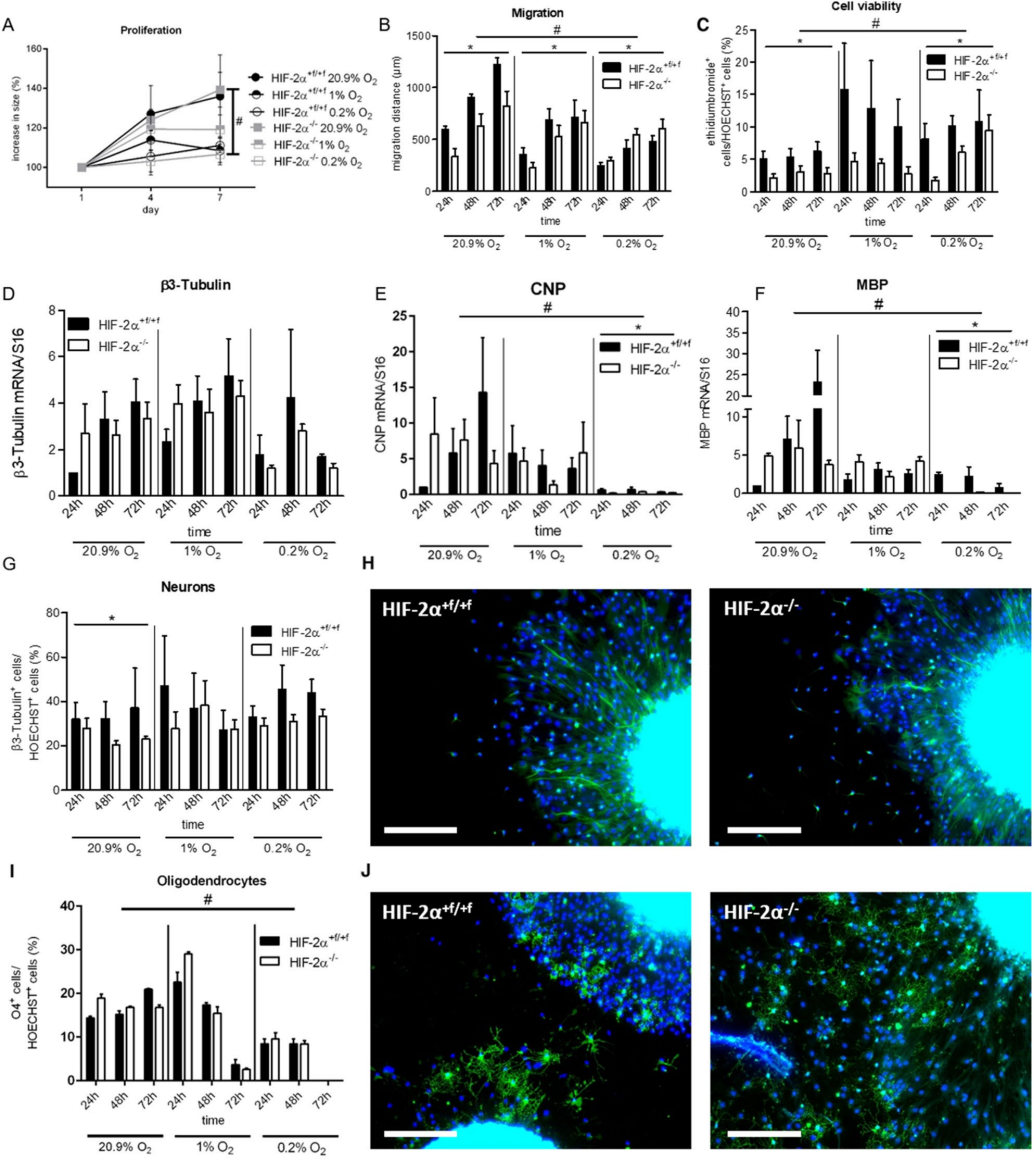
Loss of *HIF-2α* in NSCs affects various parameters during neural development in vitro. (**A)** Assessment of proliferation in spheres by measuring sphere diameter over time. (**B)** Quantification of cell migration at different time points, distance was measured from the edge of the sphere to the farthest migrated cell. (**C)** Cell viability was assessed by ethidium bromide exclusion. (**D-F)** Real-time PCR analyses for oligodendrocytes (*Mbp*) and neurons (*β(III)Tub*) were quantified and normalised for *Rsp16* expression. (**G-J)** Cells were stained with antibodies against β(III)tubulin (β(III)Tub^+^, green, **H**) for neurons and O4^+^ (green, **J**) for oligodendrocytes. Cell nuclei were counterstained with Hoechst (blue). Scale bars: 200 µm. All data are shown as mean ± SE of three independent experiments.*p < 0.05 compares *Hif-2α*^+*f/*+*f*^ and *Hif-2α *^*-/-*^ over time for a specific oxygen concentration. #*p* < 0.05 indicates general influence of oxygen levels for both phenotypes combined.

For mechanistic studies, we employed the neurosphere system^15^. Neural stem cells were isolated from new born (P0) wild type and knockout pups and cultivated as neurospheres under atmospheric O_2_ concentrations. For experiments, proliferating or differentiating neurospheres were incubated under normoxic (20.9% O_2_), hypoxic (1% O_2_) or severely hypoxic (0.2% O_2_) conditions. First, we studied proliferation in wild type and knockout spheres. Size of the spheres, which is linearly correlated with cell numbers^15^, was assessed after 1, 4, and 7 days. Over time, spheres grew under all three oxygen conditions, although proliferation was highest under normoxic and lowest under severely hypoxic conditions (Fig. 3A). No differences between wild type and knockout spheres were found. Next, to investigate migration, neurospheres were plated on a protein matrix. After mitogen withdrawal, neural cells started to migrate radially out of the spheres with increasing distances over time. Migration was quantified after 24, 48, and 72 h. In wild type cells, reduced O_2_ concentrations caused shorter migration (Fig. 3B). Interestingly, *Hif-2α*^*-/-*^ cell migration was significantly lower under normoxia compared to wild type controls but this difference was lost with increasing hypoxia. To analyses, if the reduction in migration was caused by cell death, we performed a cell viability assay. Oxygen concentration had a major impact on cell viability and induced higher cell death rates under hypoxia. At all oxygen levels, the number of dead cells in the *Hif-2α*^*-/-*^ neurospheres was lower compared to wild type cells indicating a crucial effect of HIF-2α concerning cell death (Fig. 3C). As the loss of HIF-2α led to reduced numbers of pyramidal cells in the RSC and a reduction in MBP, we analyzed differentiation into neurons and oligodendrocytes in vitro. First, we checked for changes in mRNA expression of *β(III)-Tubulin* during differentiation. We found no significant differences between wild type and knockout spheres or different oxygen concentration (Fig. 3D). However, immunocytochemical staining against β(III)-Tubulin showed a significant reduction in immunoreactive cells in HIF-2α deficient neurospheres under normoxia, but not under hypoxia or severe hypoxia (Fig. 3G and 3H). Interestingly, no morphological differences were seen. To analyze oligodendrocyte development, we looked for *Cnp* expression for OPCs and *Mbp* expression as a marker for mature oligodendrocytes and found a strong inhibition of both genes under hypoxia (Fig. 3E and F). A significant difference between knockout and wild type cells was only observed under severely hypoxic conditions, where almost no *Cnp* and *Mbp* was detectable in the knockout spheres. Yet, immunocytochemical staining against the O4 epitope revealed no differences in oligodendrocyte numbers and morphologies between wild type and knockout spheres (Fig. 3I and J), although prolonged incubation under hypoxic or severely hypoxic conditions reduced the overall number of O4^+^ cells in both genotypes.

### Deficiency in HIF-2α affects synaptogenesis

**Figure 4:**
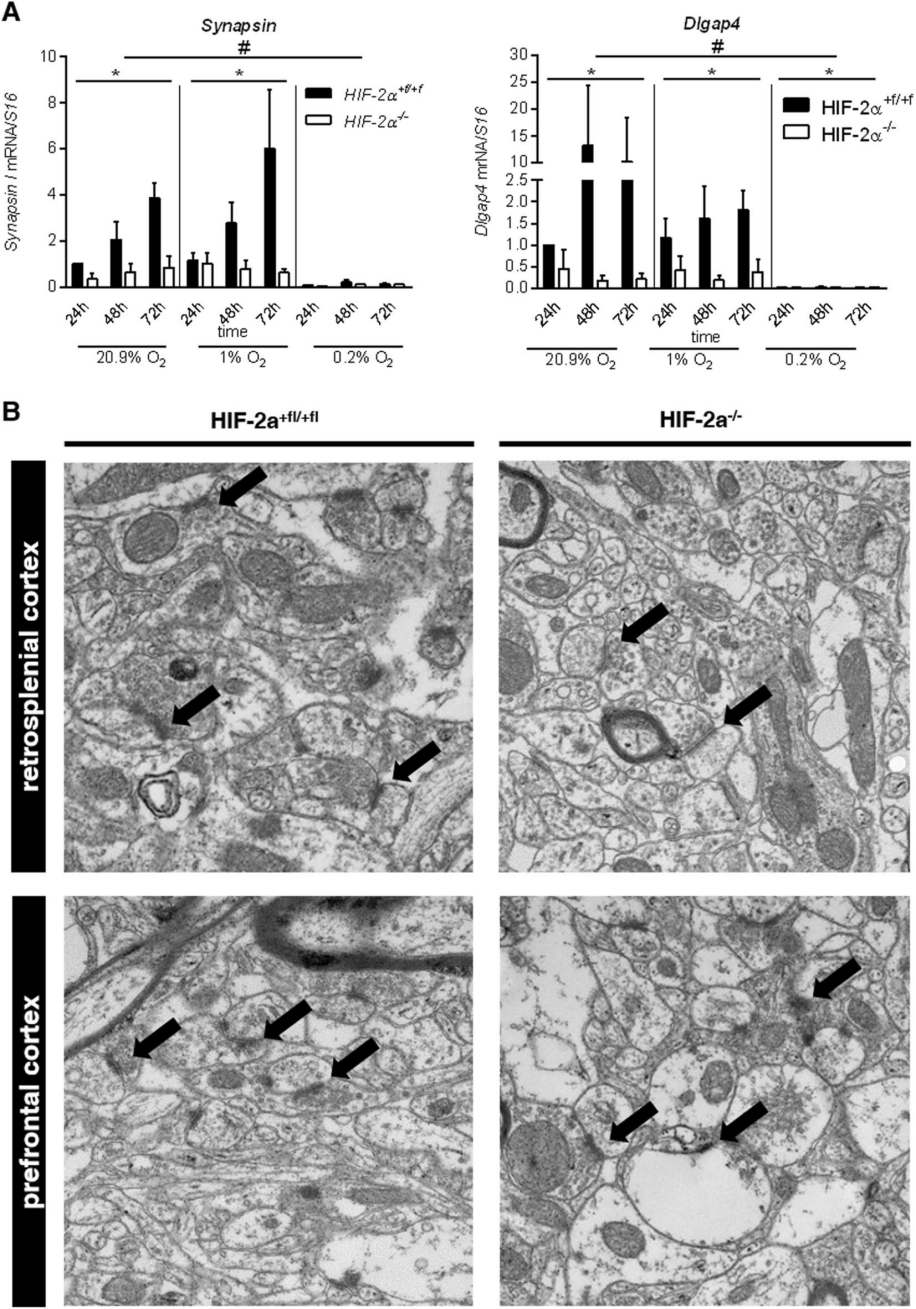
Deficiency in HIF-2α affects synaptogenesis. (**A)** Real-time PCR analyses for pre-synapses (*Synapsin I*) and post-synapses (*Dlgap4*) were quantified and normalised for *Rsp16* expression. (**B)** Representative EM photomicrographs from synapses (arrows) in the RSC and PFC of wild type and *Hif-2α* knockout animals. All data are shown as mean ± SE of three independent experiments. *p < 0.05 compares *Hif-2α*^+*f/*+*f*^ and *Hif-2α *^*-/-*^ over time for a specific oxygen concentration. #*p* < 0.05 indicates general influence of oxygen levels for both phenotypes combined.

Beside the reduced *Neurogranin* mRNA expression in the cortices of *Hif-2α*^*-/-*^ animals, we also detected a reduction in *Syn1* expression. Therefore, we measured mRNA levels of the synapse associated proteins *Syn1* and *SAP90/PSD-95-associated protein 4* (*Dlgap4*) in neurospheres under different oxygen concentrations (Fig. 4A). Spheres derived from *Hif-2α*^*-/-*^ mice showed a strong reduction in the expression of *Syn1* and *Dlgap4* under normoxia and hypoxia. Under severe hypoxia, hardly any of these mRNAs were detectable in both groups. Based on these in vivo and in vitro findings of reduced synaptic mRNA expression, we analyzed synapses in wild type and knockout mice in an electron microscope (Fig. 4B). We analyzed synapse diameter, active zone length, amounts of synaptic vesicles, and abundance of synapses. Strikingly, we did not encounter any differences in synapse morphology between the two groups neither in the RSC nor in the PFC (data not shown). Combined, we demonstrated that expression of synaptic markers was highly oxygen dependent and was down-regulated after loss of HIF-2α, whereas no differences in morphology of synapses were obvious in vivo.

### HIF-2α alters neurogenesis pathways during development

**Figure 5:**
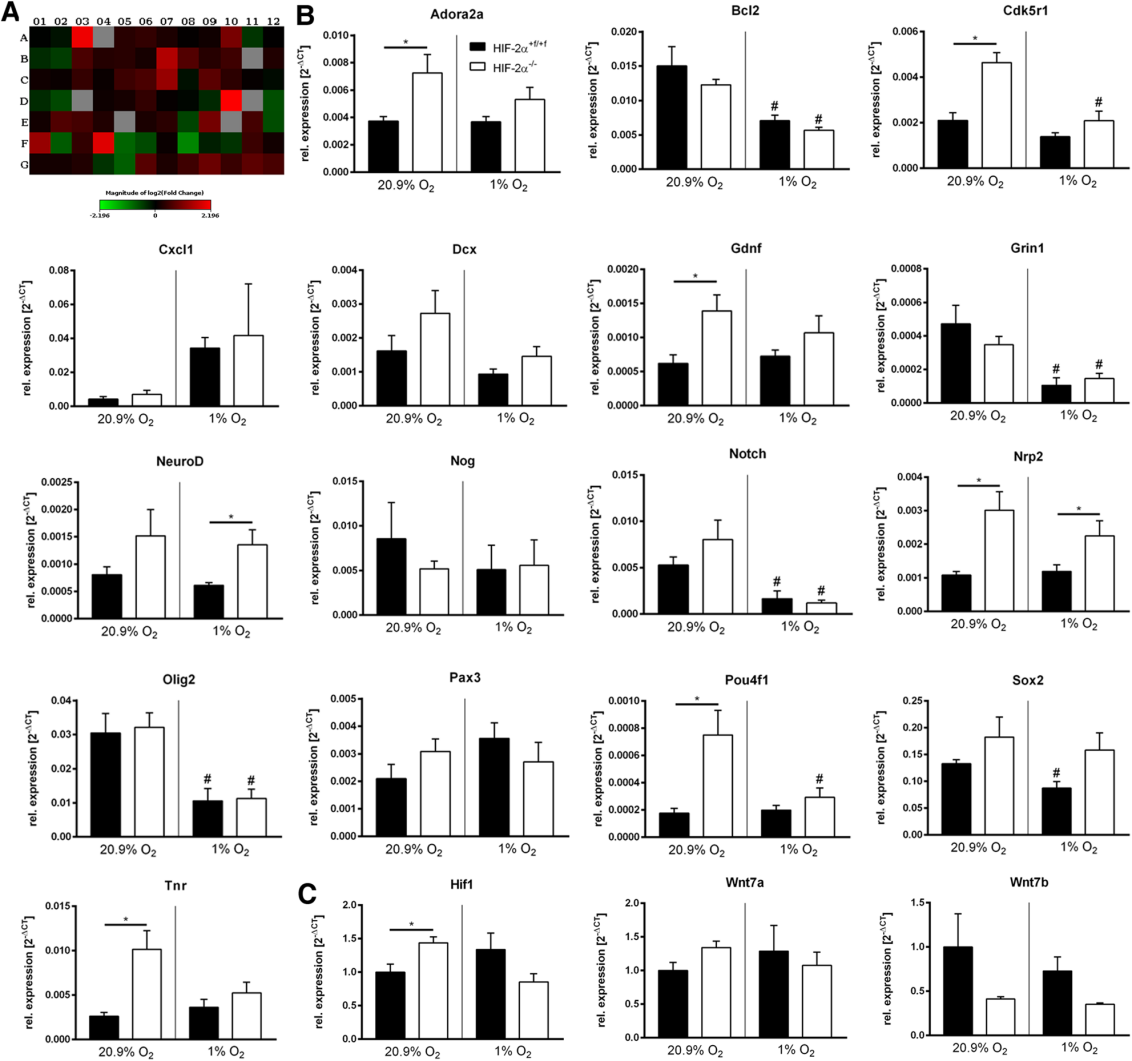
HIF-2α alters neurogenesis pathways during development. (**A)** Overview of gene expression profiles in knockout spheres compared to wild type spheres using the Qiagen RT^2^ profiler array. For individual results see supplementary Table 1. (**B)** Real-time PCR analyses for genes of interest were quantified and normalised for *Rpl13a* expression. (**C)** Real-time PCR analyses for indicated genes were quantified and normalised for *Rpl13a* expression. All data are shown as mean ± SE of three independent experiments. *p < 0.05 compared as indicated.

As HIF-2 is a transcription factor, we employed a neurogenesis pathway-focused RT^2^ Profiler Array to screen for changes in expression profiles of neurogenesis pathway genes. Spheres of wild type and knockout animals were differentiated for 24 h under hypoxia prior to mRNA isolation. The profiling hits were based on predefined criteria including gene alterations below *p-value* < 0.05 and fold changes (fc) larger than ± 2 (Supplemental Table s1). The array showed gene expression alterations in the knockout neurospheres for 9 genes, i.e. *Adenosine A2a receptor* (*Adora2a*, fc = 4.19), *B-cell leukemia/lymphoma 2* (*Bcl2*, fc = 2.05), *Chemokine (C-X-C motif) ligand 1* (*Cxcl1*, fc = 2.88), *Glial cell line derived neurotrophic factor* (*Gdnf*, fc = 2.48), *Neurogenic differentiation 1* (*Neurod1*, fc = 4.58), *Neuropilin 2* (*Nrp2*, fc = 2.00), *Oligodendrocyte differentiation factor 2* (*Olig2*, fc = 2.48), *Paired box gene 3* (*Pax3*, 3,79), and *POU domain, class 4, transcription factor 1* (*Pou4f1*, fc = -2.23). Additionally, *Cyclin-dependent kinase 5, regulatory subunit 1* (*Cdk5r1*, fc = 1.56), *Doublecortin* (*Dcx*, fc = 1.63), *Glutamate receptor, ionotropic, Nmda 1* (*Grin1*, fc = 1.71), *Noggin* (*Nog*, fc = 1.61), *Notch gene homolog 1* (*Notch1*, fc = 1.50), *SRY-box containing gene 2* (*Sox2*, fc = 1.66), and *Tenascin R* (*Tnr*, fc = 1.73) were identified as genes of interest for further evaluation (Fig. 5A). We subsequently conducted RT-qPCR validation on individual samples from normoxic and hypoxic wild type and knockout spheres on profiling hits from pooled array samples (Fig. 5B). Overall, the expression analyses demonstrated that only *Cxcl1* was up-regulated under hypoxia compared to normoxia, although the knockout samples were not significantly different to wild type spheres due to high standard deviations. A down-regulation under hypoxia was found in *Bcl2*, *Grin1*, *Notch1*, *Olig2*, and *Sox2* (only in wild type spheres). Interestingly, *Adora2a*, *Cdk5r1*, *Gdnf*, *Pou4f1*, and *Tnr* were significantly up-regulated in the knockout samples compared to wild type controls under normoxia, but not under hypoxia. Whereas *Neurod1* and *Nrp2* were up-regulated in knockout samples under normoxia as well as under hypoxia, though *Neurod1* reached significance only under hypoxia. Additionally, we analyzed expression of *Hif-1α* and the *Wingless/Integrated* (*Wnt*) pathway genes *Wnt7a* and *Wnt7b* that are key to oligodendrocyte precursor cell (OPC) maturation arrest under hypoxia^16^. *Hif-1α* mRNA was significantly increased in knockout cells and down-regulated upon hypoxia, whereas expression in wild type sample was unaffected. *Wnt7b* was decreased in knockout samples under normoxia as well as under hypoxia, whereas *Wnt7a* was not altered (Fig. 5C). Combined, we found that loss of HIF-2α leads to alterations of neurogenic pathway genes during development, especially under normoxic conditions.

## Discussion

Our study revealed the presence of HIF-2α in the adult brain of mice even under normoxic conditions. Deficiency of HIF-2α during brain development lead to a loss of pyramidal cells in the RSC, accompanied by severe impairment in learning and memory. Studies in neural stem cells to unravel the underlying mechanisms showed that the loss of pyramidal cells in the RSC was caused by deficits in migration capabilities in *Hif-2*^*-/-*^mice due to altered expression patterns of genes highly associated with neuronal migration and positioning.

HIF-1 has been found to play a crucial role during brain development, because loss of HIF-1α during development leads to a hydrocephalus accompanied by reduced neuronal cells and impaired spatial memory^9^. While we found HIF-1α positive cell only under hypoxia, HIF-2α already accumulated under normoxic conditions in the adult mouse brain. HIF-2α is known to become stable at higher oxygen levels than HIF-1α *in vitro*^17^ and has been shown to be active under mild hypoxia^18^ (~ 5% O_2_). Hence, HIF-2α appears to drive responses at more moderate levels of hypoxia or even normoxia and in forms of long-lasting hypoxia^19^. Normal oxygen levels in the brain lie between 11.4 and 53.2 mmHg^17^, which is exactly the range in which one would expect HIF-2α to be stable. Conversely, HIF-1α is most active during short periods of intense hypoxia^20^. Therefore, we hypothesize that HIF-1 protects the brain in cases of sudden hypoxia, whereas HIF-2 is essential for normal brain function and development. Interestingly, in a study by Wiesener and colleagues HIF-2α protein in the brain of rats was only found after treatment with 0.1% carbon monoxide to cause anemic hypoxia^21^. The reason for this discrepancy with our data is unclear but might be due to species-specific or methodological differences and needs to be investigated in the future.

To test this hypothesis, we bred mice with a neuro-specific *Hif-2α* knockout. These animals showed no obvious neurological defects. However, histological analysis revealed a reduced number of predominantly pyramidal cells in the RSC after loss of HIF-2α (Fig. 2). The RSC is a member of a core network of brain regions responsible for a range of cognitive functions, including episodic memory, navigation, imagination, and future planning. In this context the RSC interacts with the hippocampal formation, the para-hippocampal region, including the entorhinal cortex, and thalamic nuclei^14^. Recently, it was shown that after RSC removal in macaque monkeys, postoperative retention tests revealed a significant retrograde memory loss compared to control animals^22^. Accordingly, functional MR imaging studies of humans demonstrated that the RSC is also activated when participants are asked to recall autobiographical memories^23,24^. Here, we found that the loss of pyramidal cells in the RSC was associated with significant impairment in learning and memory as detected by the NOR test (Fig. 2). A recent study on the role of astrocytic HIF illustrates that the loss of HIF-2α in astrocytes leads to disturbances of long-term potentiation (LTP) in acute hippocampal slices, which has been connected with learning and memory^12^. Interestingly, this effect was only observed under normoxic conditions but not after hypoxic exposure, also indicating a role of HIF-2α during normoxia.

To investigate the cause of less neurons in the RSC, we employed the neurosphere assay as an in vitro model for brain development, and assessed proliferation, migration, differentiation, and apoptosis^15^. In this context, we encountered impaired migration capabilities in *Hif-2α* knockout spheres, especially under normoxic conditions. Moreover, we observed a moderately, but significantly reduced number of neurons in the migration area of knockout neurospheres under normoxia which could be caused by reduced migration capabilities of HIF-2α deficient neurons. While it is known that HIF-1α is involved in motility of various cells types, including microglia^25^, neural crest cells^26^, and neural progenitor cells^27^, a role for HIF-2α in cell motion is so far unknown. Our data showing a reduction in migration distance of around 30% due to loss of HIF-2α suggest that the specific loss of neurons in the RSC is caused by insufficient invasion into this brain area.

Dozens of functionally distinct areas exist across the cortex and differences between these functional areas are hypothesized to emerge from a molecular protomap along the germinal zone during neurogenesis^28^. To elucidate the reason for the specific migration deficit of neurons into the RSC, we screened for gene expression alterations caused by loss of HIF-2α. We could demonstrate that several genes were altered in knockout neurospheres, especially under normoxic conditions (Fig. 5). All these genes play a role in neural migration and patterning. A recent study by Nowakowski and colleagues showed spatiotemporal expressions of ADORA2A, which is strongly expressed in the PFC compared to cortical area V1, and NEUROD1, which is heterogeneously activated during radial glia diversification, indicating a role in migration and positioning of neurons for both genes^29^. GDK5R1 is a neuron-specific activator of the cyclin-dependent kinase 5 (GDK5) that is required for invading neurons to reach their final position during brain development^30^. GDNF signaling via GFRalpha1 was shown to promote the differentiation of ventral precursor cells into GABAergic neurons, enhancing their neuronal morphology and motility^31^. A study by Ng et al*.* points to a new role for NRP2 in the positioning of neurons during adult hippocampal neurogenesis^32^. POU4F1 (also known as BRN3A) knockout mice show a loss of neurons in the trigeminal ganglia, the medial habenula, the red nucleus, and the caudal region of the inferior olivary nucleus but not in the retina and dorsal root ganglia^33^. TNR is an extracellular matrix glycoprotein that is restricted to the central nervous system, where it acts as a multifunctional and versatile molecule. It was reported that the spatiotemporal distribution of TNR parallels neuronal migration^34^. According to the protomap hypothesis, molecular differences among progenitor cells subdivide the emerging cortical sheet into distinct areas. The extent to which transcriptomic differences establish distinct laminar and areal patterns is difficult to determine and needs to be determined further in future.

Synaptic transmission accounts for up to 50% of cerebral oxygen consumption. Although highly sensitive to changes in oxygen levels, it still remains to be elucidated how hypoxia affects synaptogenesis. Here, we showed that expression of synaptic markers is highly oxygen dependent and diminished upon loss of HIF-2α in vivo and in vitro even under normoxic conditions (Fig. 2E and Fig. 4A). Changes in expression patterns after hypoxia were also observed in other studies. A simple chronic hypoxia model of the snail *Lymnaea stagnalis* showed repressed *Syntaxin-1* (a membrane-bound presynaptic protein) and elevated *Vesicle-associated membrane protein-1* (*Vamp-1*, a vesicle-bound presynaptic protein) levels^35^. Perinatal hypoxia in rats on postnatal day ten (P10) led to impaired performance in long-term spatial learning and memory (as determined on P45) associated with decreases in the expression of the complex of PSD95 with NMDAR subunits^36^. Intermittent umbilical cord occlusion in calves resulted in a decreased immunoreactivity of SYN1 in the brain of preterm animals, which indicates decreased presynaptic vesicle formation^37^. So far, we were unable to detect morphological alterations of synapses in the HIF-2α^-/-^ mice (Fig. 4B). Whether HIF-2α is involved in synapse formation or regulation and if this is involved in the deficits of learning and memory needs to be investigated in the future.

The cell membrane of oligodendrocytes forms myelin sheaths providing functional and trophic support for axons in the white matter of the central nervous system. Yuen and colleagues showed in an elegant study that oxygen tension, mediated by HIF function, is an essential regulator of postnatal myelination, and that hypoxic treatment leads to a reduction of myelin formation due to an OPC maturation arrest^16^. In this study we confirmed the expansion of myelin was greatly reduced in hypoxic and severely hypoxic conditions (Fig. 3F).We assume that this was likely caused by a reduction in *Olig2* expression under hypoxia (Fig. 5), an important factor in oligodendrocyte development. Immunocytochemistry revealed that reduced *Mbp* expression was accompanied by a reduction in *Cnp* expression and in O4^+^ (pre-)oligodendrocytes. These results contradict the findings of Yuen et al*.* as they showed a maturation arrest without altered total oligodendrocyte lineage numbers^16^. However, we proved that the loss of HIF-2α leads to a reduction of MBP formation in vivo and in vitro even under normoxic conditions (Fig. 2B and 2E, Fig. 3F), although the numbers of pre-oligodendrocytes & mature oligodendrocytes were comparable in both genotypes (Fig. 3I). The loss in MBP protein might be an alternative explanation for the deficits in learning and memory in the *Hif-2α* knockout mice. However, a thorough analysis of *Mbp* heterozygous mice that show a strong reduction in *Mbp* expression, showed no differences in learning and memory^38^.

It was shown that Wnt signaling inhibits OPC maturation during development in health and disease^16,39^. Yuen et al*.* showed that constitutive HIF-1α/2α stabilization results in OPC maturation arrest through autocrine activation of canonical Wnt7a/7b^16^. Here, we found a reduced expression of *Wnt7b* after loss of HIF-2α, whereas *Wnt7a* was unaffected, indicating that Wnt7b is likely controlled by HIF-2α and Wnt7a by HIF-1α. Further studies are required to investigate the specific roles of the HIF isoforms in oligodendrocyte development.

Interestingly, all genes we showed to be altered in the *Hif-2α*^*-/-*^ deficient mice are associated with Alzheimer disease (AD) and memory loss. A gene-based association analysis identified ADORA2A associated with hippocampal volume in mild cognitive impairment and AD^40^. GDNF is down-regulated in *post-mortem* middle temporal gyrus of AD patients^41^, and GDNF administration can protect against AD-like changes induced by injection of aluminum complexes in rabbits^42^. GDK5R1 activates GDK5 that contributes to the pathophysiology of AD. CDK5 dysregulation facilitates extracellular deposition of Aβ in senile plaques and intracellular accumulation of hyperphosphorylated Tau protein in neurofibrillary tangles^43^. Directed expression of NEUROD1 in cycling hippocampal progenitors rescued memory loss in an *APP* x *PS1* mouse model of AD^44^. NRP2 knockout mice showed striking impairments in learning and memory^45^. Low-level lead exposure led to spatial learning deficits in rats due to reduced protein and mRNA levels of POU4F1^46^. TNR was found to be implicated in AD in a genome-wide association study^47^. WNT7B is down-regulated in the entorhinal cortex and the hippocampus of AD patients^48^. Finally, *Hif-2α* itself is one of the genes that is down-regulated during AD^49^. Especially striking is the discovery that the earliest metabolic decline in AD is centered on the RSC^50^, an area, which we showed to be specifically vulnerable to the loss of HIF-2α. Of note, native Tibetans in the Qinghai-Tibetan plateau, who show a special *Hif-2α* gene polymorphism playing a key role in high altitude adaptation^51^, have one of the lowest prevalence of AD in the world^52^.

Our study provides new evidence of an important function of HIF-2α in normal brain function and during development. Genes involved in neuronal migration and positioning need to be orchestrated in a highly specific spatiotemporal manner during development. The hypoxia-inducible factor as a transcription factor conducts several hundred target genes during brain development and maintains normal function of the adult brain by reacting to changes in tissue oxygen tension. Moreover, a role for HIF-2α during normal brain aging and especially during pathological degeneration needs future investigation as hypoxia is one of the key components in the pathophysiology of stroke, Parkinson's or Alzheimer's disease.

## Experimental procedures

### Animals

Inbred C57BL/6 J mice with loxP sites flanking exon 2 of the *Hif-2α* gene (*Hif-2α*^+*f/*+*f*^, purchased from The Jackson Laboratory, Bar Harbor, ME, USA) were crossbred with mice with a NESTIN (NES) promoter driving CRE recombinase (*Hif-2α*^+*f/*+*f*^* x Nes-Cre*) to achieve a neural specific *Hif-2α* knockout. Exon 2 encodes for the DNA binding site of translated HIF-2α protein. Littermates negative for CRE recombinase (*HIF-2α*^+*f/*+*f*^) served as control animals. Drinking water and standard rodent pellets were provided ad libitum. Wild type and knockout animals demonstrated physiological habitus and bred regularly. Animal breeding was performed in full accordance with the German law for animal welfare and with institutional regulations for animal breeding and handling and approved by the State Agency for Nature, Environment and Consumer Protection North Rhine-Westphalia (file reference: 84–02.04.2016.A173).

### Cell culture

Murine progenitor cells were isolated from the cortex of postnatal (P0) wild type and *Hif-2α*^*-/-*^ mice and cultivated as neurospheres. Brains were dissected and transferred to minimal essential medium (MEM; Thermo Fisher, Waltham, MA, U.S.A.). Meninges, hippocampi, and olfactory bulbs were removed, and the cerebral cortices were isolated. Subsequently, the tissue was enzymatically digested with 30 U/mL papain (Worthington, Freehold, NJ, U.S.A.) for 20 – 30 min at 37° C to obtain single cell suspensions. Enzyme activity was stopped by adding 1 ml of ovomucoid [1 mg/ml trypsin inhibitor (Merck, Darmstadt, Germany), 50 μg/ml BSA, and 40 μg/ml DNaseI (Worthington) in MEM). After centrifugation at 1000 g for 5 min, cell pellets were resuspended in neurosphere medium consisting of DMEM/F-12 (1:1, Thermo Fisher) containing 0.2 mg/ml l-glutamine (Merck, Darmstadt, Germany) and 2% v/v B27 supplement (Thermo Fisher), 100 U/ml penicillin, and 100 μg/ml streptomycin (both Merck). Cells were allowed to form free-floating spheres at 37° C in 20.9% O_2_/5% CO_2_ at a density of 10^5^ cells/ml in T25 flasks (bulk culture) in neurosphere medium in the presence of 20 ng/mL epidermal growth factor (EGF) and basic fibroblast growth factor (bFGF, Preprotech), Every 2–3 days half of the culture medium was changed and supplemented with 20 ng/mL EGF and bFGF. After 7 days in vitro (div) spheres were used for further experiments.

### Proliferation assay

For proliferation analyses individual spheres were cultivated in 96-well plates (bulk culture) in neurosphere medium containing 20 ng/mL EGF and bFGF and incubated under normoxia (20.9% O_2_), hypoxia (1% O_2_), and anoxia (0.2% O_2_). Sphere diameter was measured on days 0, 3, and 7 to determine proliferation, as the diameter correlates directly to the cell number inside the sphere^15^. Moreover, to define the number of proliferating cells, cells were labelled with CellTiter-Blue assay (Promega, Madison, WI, USA) according to manufacturer’s instructions, also on days 0, 3, and 7.

### Migration and apoptosis assay

Migration and differentiation was initiated by mitogen withdrawal. Therefore, spheres were plated in 4-well dishes (Greiner Bio One, Kremsmuenster, Austria), coated with 10 μg/ml poly-ornithine and 10 μg/ml laminin-1 (Merck) in neurosphere medium containing 1% v/v FCS (Merck). Migration distance was assessed after 24, 48, and 72 h under normoxic, hypoxic or anoxic conditions by measuring the distance from the edge of the sphere to the farthest migrated cells at four defined positions per sphere.

Subsequently, the number of dead cells was determined by double staining cells with 2% w/v ethidium bromide (Merck) and 0.1 µg/mL Hoechst 33,258 (Merck) for 2 min. For analysis, spheres were examined using a fluorescence microscope (Axiovert 200 m, Zeiss Jena, Germany) and stained cells were counted manually in relation to the total number of nuclei in the field of view.

### Differentiation assay

After differentiation for 24, 48, and 72 h under normoxic, hypoxic, and severely hypoxic conditions, cells were fixed in 4% w/v paraformaldehyde (Merck) for 15 min at room temperature. Subsequently, immunocytochemistry was performed as previously described with various antibodies^15^ (see Table 1).

**Table 1:** Primers.

Primers	5’	3’
*Mbp*	CCC CAG AGC TGG TGC TTT TA	GAG AAC TCC TGC AGT CCC AC
*Nrgn*	CCC AGC ATC GTA CAA ACC CA	GGC GCT CTC CGT GCA G
*Dlgap4*	GGC CAT GAT CAA CAG GTC CG	CCT CTG CGG TTG TAG ACT CG
*SynI*	CCC AGC TCA ACA AAT CCC AGT	TCA CCT CGT CCT GGC TAA GG
*β(III)- Tub*	TTT TCG TCT CTA GCC GCG TG	GAT GAC CTC CCA GAA CTT GGC
*Adora2a*	GCC ATC CCA TTC GCC ATC A	GCA ATA GCC AAG AGG CTG AAG A
*Bcl2*	ATG CCT TTG TGG AAC TAT ATG GC	GGT ATG CAC CCA GAG TGA TGC
*Cdk5r1*	CTG TCC CTA TCC CCC AGC TAT	GGC AGC ACC GAG ATG ATG G
*Cxcl1*	CTG GGA TTC ACC TCA AGA ACA TC	CAG GGT CAA GGC AAG CCT C
*Dcx*	CAT TTT GAC GAA CGA GAC AAA GC	TGG AAG TCC ATT CAT CCG TGA
*Gdnf*	CCA GTG ACT CCA ATA TGC CTG	CTC TGC GAC CTT TCC CTC TG
*Grin1*	AGA GCC CGA CCC TAA AAA GAA	CCC TCC TCC CTC TCA ATA GC
*NeuroD1*	ATG ACC AAA TCA TAC AGC GAG AG	TCT GCC TCG TGT TCC TCG T
*Nog*	GCC AGC ACT ATC TAC ACA TCC	GCG TCT CGT TCA GAT CCT TCT C
*Notch1*	GAT GGC CTC AAT GGG TAC AAG	TCG TTG TTG TTG ATG TCA CAG T
*Nrp2*	GCT GGC TAC ATC ACT TCC CC	CAA TCC ACT CAC AGT TCT GGT G
*Olig2*	TCC CCA GAA CCC GAT GAT CTT	CGT GGA CGA GGA CAC AGT C
*Pax3*	TTT CAC CTC AGG TAA TGG GAC T	GAA CGT CCA AGG CTT ACT TTG T
*Pou4f1*	CGC GCA GCG TGA GAA AAT G	CGG GGT TGT ACG GCA AAA TAG
*Sox2*	GCG GAG TGG AAA CTT TTG TCC	CGG GAA GCG TGT ACT TAT CCT T
*Tnr*	GGC TGG AGG TGA CTA CAG AAA	GAA GAC CAT AGG CTG TTC CTT G
*Hif-1α*	ACC TTC ATC GGA AAC TCC AAA G	CTG TTA GGC TGG GAA AAG TTA GG
*Wnt7a*	GAC AAA TAC AAC GAG GCC GT	GGC TGT CTT ATT GCA GGC TC
*Wnt7b*	TCT CTG CTT TGG CGT CCT CTA C	GCC AGG CCA GGA ATC TTG TTG
*Hif-2α exon 2*	AGG AGA CGG AGG TCT TCT ATG A	ACA GGA GCT TAT GTG TCC GA
*Hif-2α exon 11*	GCC CTA CTA AGT GGC CTG TG	GGA GGT TCC AAC TGC GAT GA

### Polymerase chain reaction

Total RNA was isolated from murine brains and neurospheres with the RNeasy Mini Kit (Qiagen, Hilden, Germany) or the NucleoSpin RNA kit (Macherey–Nagel, Dueren, Germany). RT-PCR was performed as previously described with SYBR green fluorescent dye (Eurogentec, Verviers, Belgium) and the iQ5/C1000 Real-time PCR Detection System (Bio-Rad Laboratories GmbH, Munich, Germany). Amounts of complementary DNA were amplified with gene specific primers (see Table 2) and normalized to ribosomal protein (*Rsp16*) or *60S ribosomal protein L13a* (*Rpl13a*) as indicated. Expression was calculated with the 2^-ΔΔCT^ method.

**Table 2:** Antibodies.

Visualisation of	Primary antibody	Secondary antibody
Astrocytes	Glial fibrillary acidic protein (GFAP) – (mouse, #MAB360, Millipore)	M.O.M.™ anti-mouse IgG biotinylated – (#BMK2202. Vector Laboratories)
HIF-1α	Hypoxia-inducible factor-1α – (rabbit, #10,006,421, Cayman Chemicals)	Anti-rabbit biotinylated –(#E043201, DAKO)
HIF-2α	Hypoxia-inducible factor-2α – (rabbit, #NB100-122, Novus)	Anti-rabbit biotinylated –(#E043201, DAKO)
Oligodendrocytes	Myelin basic protein (MBP) – (rabbit, #M3821, Sigma-Aldrich)	Anti-rabbit biotinylated –(#E043201, DAKO)
Oligodendrocytes	Oligodendrocyte Marker O4 – (mouse, # MAB1326, R&D Systems)	Alexa 488 Goat-anti-mouse – (#A11001, Invitrogen)
Neurons	Β(III)-Tubulin (rabbit, T2200, Sigma-Aldrich)	Alexa 488 Goat-anti-rabbit – (#A11034, Invitrogen)
Pyramidal cells	Neurogranin (NRGN) – (rabbit, #10,440–1-AP, proteintech™)	Anti-rabbit biotinylated –(#E043201, DAKO)

The data supporting the findings of this study are available from the corresponding author upon request.

Genomic DNA for genotyping was isolated from murine tissue with the DNeasy Blood and Tissue Kit (Qiagen) according to the manual, and conventional RT-PCR was performed.

The Qiagen Profiler RT^2^ Array was performed according to manual. Briefly, wild type and knockout neurospheres were differentiated for 24 h under atmospheric conditions (20.9% O_2_). Subsequently, RNA was isolated and array was performed.

### Histology

Brain hemispheres of P70 mice were isolated, fixed in 4% paraformaldehyde, embedded in paraffin, and cut on a microtome (Thermo Fisher). Resulting 2 µm sections for immunohistochemistry and 7 µm sections for conventional histology were H.E. or Nissl stained and microscopically analyzed to determine morphological differences (i.e. cortex thickness at thickest point) between wild type and knockout mice. For further evaluation, immunohistochemical stainings were implemented. We applied the diaminobenzidine method with various antibodies (see Table 1). As immunoperoxidase detection system we used the Vectastain ABC-Kit (Vector Laboratories Ltd). HIF-1α and HIF-2α immunostaining was accomplished using the CSA-I Kit (DAKO).

### In situ* hybridization*

RNA in situ hybridization was performed on 4 µm thick FFPE sections of mouse brain using the RNAscope 2.5 HD Assay-brown (Advanced Cell Diagnostics) according to user manuals 322,452-USM and 322,310-USM using standard conditions. *Hif-1α* RNA was detected by RNAScope probe *Mm-Hif1a* (Cat No. 313821), *Hif-2α* by *Mm-Epas1* (Cat No. 314371). For quantification we applied the RNAscope SpotStudio v1.0 Software (Advanced Cell Diagnostics).

### Electron microscopy

For EM studies of synapses, the skull of P70 mice was opened after decapitation and brains were directly fixed in the skull using 2.5% glutaraldehyde in PBS (Merck). The RSC region and the prefrontal cortex were cut into 1 mm thick sections and transferred into 2.5% glutaraldehyde in PBS for 4 h for further fixation. Cubes (1 × 1x1 mm) of the RSC and PFC were cut and post-fixed with 1% osmium tetroxide,dehydrated through a graded series of ethanol, and embedded in Epon 812 (Shell). On a Reichert-Jung ultramicroscope 60 nm ultrathin sections were cut and placed on a copper grid. After staining with 1% uranyl acetate and 0.4% lead citrate sections were examined and digitally acquired on a transmission electron microscope (JEM 1400 Plus; JEOL, Tokyo, Japan).

### Behavioral studies

For behavioral studies, P70 mice were subjected to different tests with at least 48 h break between tests. All tests took place in a quiet, uncolored room. Animal experiments were performed in full accordance with the German law for animal welfare and with institutional regulations for animal breeding and handling and approved by the State Agency for Nature, Environment and Consumer Protection North Rhine-Westphalia (file reference: 84–02.04.2016.A173).

The novel object recognition test was used to evaluate the memory, the affinity to the unknown, the integration of different brain regions, and learning abilities. Testing was carried out in the home cage, therefore the habituation phase was omitted. Each mouse was shortly removed from its home cage, while two identical objects were positioned in the cage, and gently placed back, with the nose facing the middle point of the wall away from objects and let explore for 5 min. After a 30 min retention interval, one of the objects was replaced with a novel object and the mouse was placed in a similar manner in the cage for the 5 min exploration session. Time spent with the familiar and novel object was recorded and the discrimination and recognition ratio for the testing session was calculated for each mouse using the following formula:

$$Discrimination\;index = \frac{{\left( {Time\;exploring\;novel\;object - time\;exploring\;familiar\;object} \right)}}{{\left( {Time\;exploring\;novel\;object + time\;exploring\;familiar\;object} \right)}}$$

$$Recognition\;index = \frac{Time\;exploring\;novel\;object}{{\left( {Time\;exploring\;novel\;object + time\;exploring\;familiar\;object} \right)}}$$

Motor skills were examined on the Rotarod (RotaRod Advanced, TSE Systems GmbH, Germany). Rotation speed accelerated from 4 to 20 RPM. The experiment was stopped as soon as all four paws of the mouse touched the ground and fall latencies were measured.

To explore the edge between anxiety and curiosity the black/white-box test was employed. Each mouse was placed in the light chamber facing the glass front and its back to the dark chamber. During 5 min exploration time the following parameters were measured: period until first entrance into dark chamber, total time spent in the light chamber, and number of crossings from light to dark chamber.

### Hypoxic treatment

For HIF-1α protein stabilization, mice were exposed to a hypoxic environment. Therefore, oxygen concentration was gradually decreased in a hypoxic chamber (Baker Ruskin, Sanford, ME, USA) from normoxia (20.9%) to 10% oxygen concentration (2% down every 2 h). After 6 h at 10% O_2_, mice were euthanized and brains were isolated and fixed in the hypoxic chamber.

### Statistical analyses

In vivo experiments were analyzed by using GraphPad Prism 5 Version 5.04 (Graph-Pad Software, Inc., CA, USA, www.graphpad.com). Statistical significance was determined using unpaired, 2-tailed Student`s t-Test. For all quantified data, mean ± SEM values are presented. To calculate significance of in vitro experiments a general linear mixed model in SAS v.9.4 (The SAS Institute, Cray, NC, USA) was used to determine the relations between all measured values and between neurospheres in normoxia, hypoxia, and severe hypoxia after 24, 48, and 72 h. That includes both the difference between knockout and wild type within one oxygen concentration as well as the influence of the oxygen concentration on all groups.

## Supplementary information

Supplementary Information.
