# Supplementary material for: Hypoxia-inducible factor-2α is crucial for proper brain development
Source: Sci Rep. 2020 Nov 5;10:19146. doi: 10.1038/s41598-020-75838-4 (PMC7644612; doi:10.1038/s41598-020-75838-4)
Supplement: Supplementary file 1 — Supplementary Information. [file 41598_2020_75838_MOESM1_ESM.pdf]

Hypoxia-inducible Factor-2α is Crucial for Proper Brain Development

Kira Kleszka[1], Tristan Leu[1], Theresa Quinting[1], Holger Jastrow[2], Sonali Pechlivanis[3], Joachim Fandrey[1*], Timm Schreiber[1]

[1]Institute of Physiology, University Duisburg-Essen, Essen, Germany

[2]Institute of Anatomy & Institute for Experimental Immunology and Imaging, Imaging Centre Essen, Electron microscopy unit, University Hospital of Essen, Essen, Germany

[3]Institute for Medical Informatics, Biometry and Epidemiology, University Hospital of Essen, University Duisburg-Essen, Essen, Germany

*corresponding author

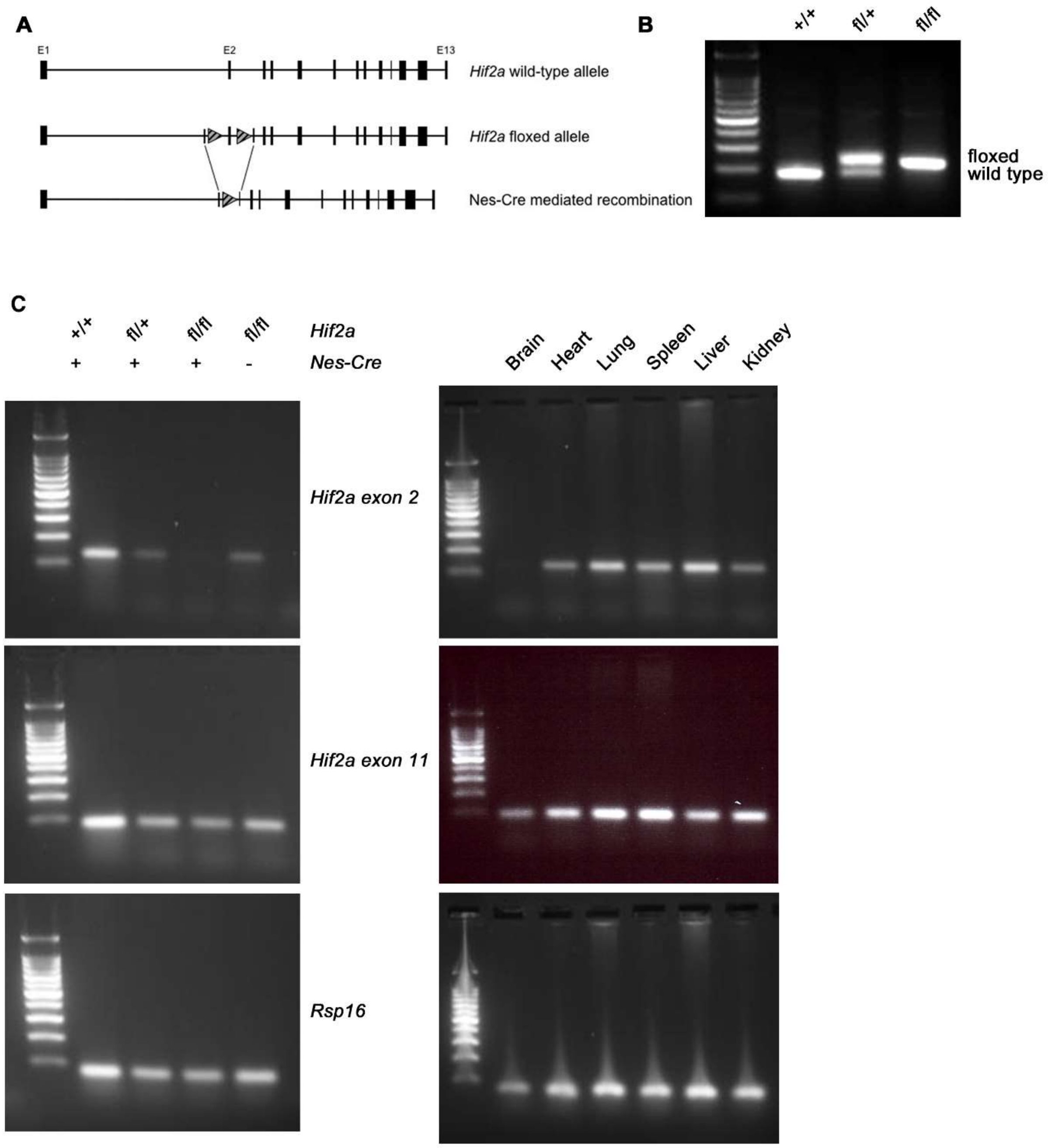

Supplementary Figure 1. A. Genetic background of the *Hif-2α* knockout mice. **B.** RT-PCR of the floxed alleles of *Hif*-2α exon 2. **C.** PCR from genomic DNA of *Hif-2α* exon 2 and exon 11, and *Rsp16* in the brain in different genetic backgrounds (left) and in different organs (right).

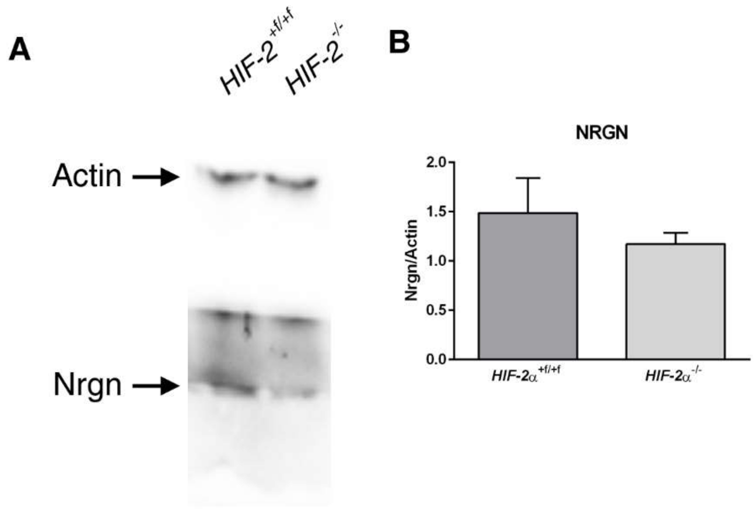

Supplementary Figure 2. A. Western blot analysis of Nrgn and actin protein from the cortices of wild type and *Hif-2α* knockout mice. **B.** Quantification of Nrgn expression normalised to actin. Data are shown as mean ± SE of three independent experiments. *$p<0.05$.

Supplementary Table 1

	**1**	**2**	**3**	**4**	**5**	**6**	**7**	**8**	**9**	**10**	**11**	**12**
A	*Ache*	*Adora1a*	*Adora2a*	*Alk*	*Apbb*	*Apoe*	*App*	*Artn*	*Ascl1*	*Bcl2*	*Bdnf*	*Bmp2*
	-1.01	-1.11	4.19	-1.59	1.27	1.34	1.18	1.03	1.09	2.05	-1.36	1.01
B	*Bmp4*	*Bmp8a*	*Cdk5r1*	*Cdk5rap2*	*Chrm2*	*Creb1*	*Cxcl1*	*Dcx*	*Dlg4*	*Dll1*	*Drd2*	*Dvl3*
	-1.28	-1.43	1.56	1.23	1.27	1.14	2.88	1.63	1.26	1.44	-1.59	1.21
C	*Efnb1*	*Egf*	*Ep300*	*Erbb2*	*Fgf2*	*Flna*	*Gdnf*	*Gpi*	*Grin1*	*Hdac4*	*Hes1*	*Hey1*
	1.10	1.06	1.36	1.06	1.43	1.56	2.48	-1.11	1.71	1.30	1.03	1.07
D	*Hey2*	*Heyl*	*Il3*	*Mdk*	*Mef2c*	*Kmt2a*	*Map2*	*Ndn*	*Ndp*	*NeuroD1*	*Neurog1*	*Neurog2*
	-1.29	-1.53	-1.59	1.09	1.14	1.34	1.03	-1.04	-1.27	4.58	-1.59	-1.59
E	*Nf1*	*Nog*	*Notch1*	*Notch2*	*Nr2e3*	*Nrcam*	*Nrg1*	*Nrp1*	*Nrp2*	*Ntf3*	*Ntn1*	*Tenm1*
	1.12	1.61	1.50	1.21	-1.59	1.17	1.28	-1.50	2.00	-1.59	1.50	-1.58
F	*Olig2*	*Pafah1b1*	*Pard3*	*Pax3*	*Pax5*	*Pax6*	*Pou3f3*	*Pou4f1*	*Ptn*	*Rac1*	*Robo1*	*Rtn4*
	2.48	-1.70	1.36	3.79	-1.81	-1.55	1.06	-2.23	-1.20	-1.35	1.41	1.12
G	*S100a6*	*S100b*	*Shh*	*Sli2*	*Sod1*	*Sox2*	*Sox3*	*Stat3*	*Tgfb1*	*Th*	*Tnr*	*Vegfa*
	1.13	1.13	1.21	-1.35	-1.75	1.66	1.18	1.44	1.88	1.39	1.73	1.53

Results of the Qiagen RT^2 profiler array for neural development in knockout spheres compared to wild type spheres after 24 hours of differentiation under 1% O_2.

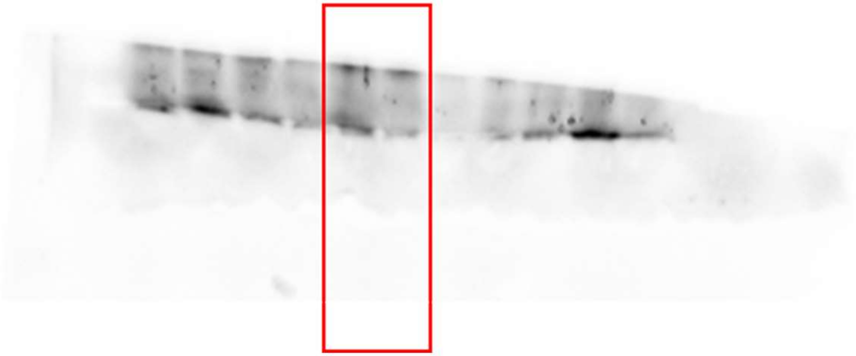

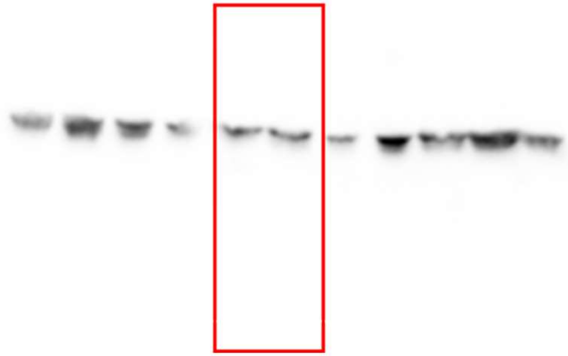

Original uncropped files from Sup Fig 2. The membrane was cut in half and the lower part was used for detection of Neurogranin (left) and the upper part for actin (right).
